# Short‐term hypoxia does not promote arrhythmia during voluntary apnea

**DOI:** 10.14814/phy2.14703

**Published:** 2021-01-10

**Authors:** Stephen A. Busch, Sean van Diepen, Richard Roberts, Andrew R. Steele, Lindsey F. Berthelsen, Megan P. Smorschok, Cody Bourgoin, Craig D. Steinback

**Affiliations:** ^1^ Neurovascular Health Laboratory Faculty of Kinesiology, Sport, and Recreation University of Alberta Edmonton Canada; ^2^ Department of Critical Care and Divison of Cardiology Department of Medicine University of Alberta Edmonton Canada; ^3^ Department of Physiology Faculty of Medicine & Dentistry University of Alberta Edmonton Canada

**Keywords:** apnea, arrhythmia, bradycardia, chemoreflex, hypoxia, vagal

## Abstract

The presence of bradycardic arrhythmias during volitional apnea at altitude may be caused by chemoreflex activation/sensitization. We investigated whether bradyarrhythmic episodes became prevalent in apnea following short‐term hypoxia exposure. Electrocardiograms (ECG; lead II) were collected from 22 low‐altitude residents (*F* = 12; age=25 ± 5 years) at 671 m. Participants were exposed to normobaric hypoxia (Spo_2_ ~79 ± 3%) over a 5‐h period. ECG rhythms were assessed during both free‐breathing and maximal volitional end‐expiratory and end‐inspiratory apnea at baseline during normoxia and hypoxia exposure (20 min [AHX]; 5 h [HX5]). Free‐breathing HR became elevated at AHX (78 ± 10 bpm; *p* < 0.0001) and HX5 (80 ± 12 bpm; *p* < 0.0001) compared to normoxia (68 ± 10 bpm), whereas apnea caused significant bradycardia at AHX (nadir end‐expiratory −17 ± 14 bpm; *p* < 0.001) and HX5 (nadir end‐expiratory −19 ± 15 bpm; *p* < 0.001), but not during normoxia (nadir end‐expiratory −4 ± 13 bpm), with no difference in bradycardia responses between apneas at AHX and HX5. Conduction abnormalities were noted in five participants during normoxia (Premature Ventricular Contraction, Sinus Pause, Junctional Rhythm, Atrial Foci), which remained unchanged during apnea at AHX and HX5 (Premature Ventricular Contraction, Premature Atrial Contraction, Sinus Pause). End‐inspiratory apneas were overall longer across conditions (normoxia *p* < 0.05; AHX *p* < 0.01; HX5 *p* < 0.001), with comparable HR responses to end‐expiratory and fewer occurrences of arrhythmia. While short‐term hypoxia is sufficient to elicit bradycardia during apnea, the occurrence of arrhythmias in response to apnea was not affected. These findings indicate that previously observed bradyarrhythmic events in untrained individuals at altitude only become prevalent following chronic hypoxia specificlly.

## Graphical abstract

In this study, voluntary apnea was used to determine whether significant chemoreceptor sensitivity develops following 5 h of normobaric hypoxia, as evident through significant bradycardia and arrhythmogenesis. The results showed that bradycardia and the incidence of arrhythmogenesis was similar between initial hypoxia exposures, as well as after 5 h of exposure. These findings demonstrate previously documented bradyarrhythmogenesis is a unique phenomenon to chronic hypoxia exposure (i.e., several days residency at altitude).

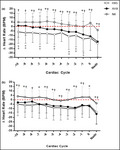

## 1 INTRODUCTION

Autonomic control of the heart relies on the interaction between sympathetic and vagal innervation, allowing for both chronotropic and inotropic regulation of cardiac function during rest and heightened physiological stress. Although sympathovagal balance has classically been described as reciprocal (Burgh Daly, 1997; Kollai & Koizumi, 1979; Paton et al., 2005), concurrent elevations in both sympathetic and parasympathetic activity can occur during periods of significant physiological stress (Kollai & Koizumi, 1979; Shattock & Tipton, 2012).

Conflict between sympathetic and parasympathetic signaling in the heart may promote cardiac events, including arrhythmogenesis (Burgh Daly et al., 1979; Paton et al., 2005; Tipton, 1989), in healthy populations exposed to unique environmental stresses. An example is the mounting evidence demonstrating an increased risk of arrhythmogenesis in lowlander dwelling populations traveling at high altitude. In particular, periods of sleep apnea that are well documented during sleep at high altitude (Ainslie et al., 2013) have previously been characterized by significant bradycardia alongside the presence of transient cardiac arrhythmias (Boos et al., 2017; Cummings & Lysgaard, 1981; Hashimoto et al., 1992; Malconian et al., 1990), indicating greater autonomic influence on the heart compared to that observed at low altitude. The oxygen‐sensitive carotid chemoreceptors have previously been implicated in these cases, as changes in heart rate observed are correlated with the hypoxic ventilatory response (Masuyama et al., 1990). More recently, our group has demonstrated the development of transient bradyarrhythmias at altitude similar to those observed during sleep via maximal volitional apnea following several days residency above 4300 m (Busch et al., 2018; Busch et al., 2020). These events were abolished through supplemental oxygen (Fraction of inspired oxygen [FiO_2_] ~1.00) (Busch et al., 2018). The findings from these previous expeditions suggest increased peripheral chemoreceptor activation during acclimatization (Sato et al., 1992) may promote autonomic conflict and lead to arrhythmias during periods of apnea.

It is well established that the carotid bodies adapt to chronic hypoxia through changes in morphology (Arias‐Stella and Valcarcel, (1976); Lahiri et al., 2000; Lopez‐Barneo et al., 2008; Wang et al., 2008), alongside increased carotid body sensitivity to hypoxia (Dempsey et al., (2014); Vizek et al., 1987) that facilitates ventilatory acclimatization under both awake and sleep states at altitude (Berssenbrugge et al., 1984; Powell et al., 1998; Sato et al., 1992). However, many of these morphological adaptations develop following several days to weeks of exposure (Iwasaki et al., (2006); Rupp et al., 2013). Furthermore, these changes do not explain the initial progressive increase in carotid body activation observed previously within animal models (Barnard et al., 1987,; Bisgard et al., 1986; Nielsen et al., 1988) that suggest peripheral chemoreceptor sensitization occurs early on under hypoxia exposure. Previous direct measures within goats have demonstrated progressive carotid sinus nerve activation to isocapnic hypoxia may even occur as early as the first 3 to 5 h (Bisgard et al., (1986); Nielsen et al., 1988). Thus, there is a mechanistic basis for hypothesizing initial carotid sinus sensitization observed during short‐term hypoxia exposure may begin to evoke chemoreflex‐mediated bradyarrhythmogenesis earlier on than that observed under apneic conditions at altitude, though no studies have currently explored this. Therefore, our goal was to investigate whether vagal mediated bradycardia or conduction abnormalities previously observed during wakefulness at altitude (Busch et al., 2018; Busch et al., 2020) begin to develop during short‐term (~5 h) hypoxia exposure. We hypothesized that short‐term hypoxia exposure would begin to unmask heightened vagal activity as an indirect marker of initial carotid sinus sensitization to steady‐state poikilocapnic hypoxia. In addition, this heightened vagal drive would become further evident during volitional apnea as characterized by significant bradycardia and arrhythmogenesis.

## 2 METHODS

### 2.1 Study participants

Twenty‐two participants (12 females; age=25 ± 5 years) were tested after providing informed written consent. Female participants were either tested within 7 days following cessation of menses or were on oral contraception and were tested during their placebo phase. All participants were healthy, and self‐reported no known history of abnormal heart function, cardiovascular, respiratory, or nervous system disease. Participants were not on any medication that affected cardiac autonomic regulation, nor had they been to altitude (>2500 m) within the 3 months prior to testing. All procedures were approved by the University of Alberta Biomedical Research Ethics Board, (Pro00073518) in compliance with the declaration of Helsinki.

### 2.2 Instrumentation

Testing took place at the University of Alberta (Edmonton, Canada ~698 m). All participants had fasted for at least 12 h prior to testing and had been requested to abstain from alcohol, caffeine, and strenuous exercise prior to testing.

Participants were tested in the supine position. Continuous ECG (Lead II) and arterial blood pressure (finger photoplethysmography; Finometer Pro, Finapres Medical Systems) were collected at 1 kHz (ADInstruments, Chart Pro v8.3.1). The brachial arterial pressure waveform was back calibrated through return‐to‐flow correction confirmed against manual brachial measurements. Mean (MAP), systolic (SBP) and diastolic (DBP) pressures were calculated on a beat‐by‐beat basis from the calibrated pressure waveform. Beat‐by‐beat cardiac output (CO) was calculated using the Model Flow algorithm and used to calculate total peripheral resistance (TPR=MAP/CO). SpO_2_ was continually assessed throughout the 5‐h exposure (pulse oximetry; Nellcor N‐600x, Medtronics).

Participants were outfitted with an oronasal facemask (7400 Series V Silicon, Hans Rudolph; dead space 40.2–49.1 ml) that had an affixed two‐way non‐rebreathing valve (Y‐shape series 2730, Hans Rudolph; dead space 100.5 ml). The inspiratory valve port was further attached to a three‐way valve (T‐shape Series 7100, Hans Rudolph dead space 20.4 ml) that could be switched between room air (FiO_2_ 0.2093; Pb 701 ± 3 Torr) and a 60 L non‐diffusing gas bag (R6060 Douglas Bag, Vacumed) containing a normobaric hypoxia gas mixture. Hypoxia during the 5‐h exposure was achieved via nitrogen compressor (Altitudetech 8850P, USA [FiO_2_ ~0.145]; maximum flow rate 120 L/min) that was supplemented with additional nitrogen (Praxair, Canada, FiN_2_ ~1.00) in order to reach the desired SpO_2_ (~80%; FiO_2_ range ~0.115–0.095). Output gases from the nitrogen compressor and nitrogen tank were continuously mixed (ADI Mixing Chamber; capacity 4.7 L) prior to filling the 60 L non‐diffusing gas throughout the entire 5‐h protocol.

### 2.3 Study protocol

Following instrumentation, participants lay supine for a 10‐min period under normoxic conditions. Participants then performed two maximal volitional apnea maneuvers. To account for the contribution of pulmonary stretch on autonomic outflow (Burgh Daly, 1997; Busch et al., 2019; Steinback et al., 2010), apneas were performed in a randomized fashion at either the end of tidal inspiration (“end‐inspiratory”) or at end‐tidal expiration (functional residual capacity; “end‐expiratory”). Both apnea protocols were kept within an individual's tidal volume to minimize sympathetic potentiation through baroreflex unloading at larger lung volumes (Heusser et al., 2009). Subjects were instructed to hold their breath with a closed glottis for “as long as possible” during each protocol until volitional breakpoint, but were also instructed not to “bear‐down” in a Valsalva maneuver. Both apnea duration and the volitional breakpoint of each subject were visually confirmed through cessation and return of respiratory traces. Each apnea was separated by a 2‐min recovery period, or until cardiorespiratory parameters returned to baseline values.

After normoxic apnea, attempts were performed and all measured variables returned to preapneic levels; participants were switched over to breathing the hypoxic gas mixture. FiO_2_ was altered on a case‐by‐case basis (via titration of nitrogen) between participants throughout the 5‐h protocol in order to maintain the desired steady‐state SpO_2_ (~80%); which was comparable to SpO_2_ values observed in our previous studies at altitude (Busch et al., 2018; Busch et al., 2020). Participants were monitored during the 5‐h hypoxic period for the onset of symptoms of acute mountain sickness during testing (Roach et al., 2018). Following stabilization of SpO_2_, participant free‐breathing data were collected and maximal end‐inspiratory and end‐expiratory apneas were performed at 20 min (AHX) and 5 h (HX5) during the hypoxia exposure. Between 20 min and 5 h, participants were able to lay in a semi‐recumbent position without any significant exertion while also not falling asleep. Manual blood pressures were taken at 1, 2, 3, and 4.5 h from the beginning of participants being switched over to the hypoxic gas mixture. Participants requiring bathroom breaks remained instrumented to their original breathing‐circuit (two‐way oronasal facemask attached to a Douglas‐bag containing the same hypoxic gas mixture they were initially breathing). Modifications were made to allow monitoring of SpO_2_ values during travel to‐and‐from the bathroom, including the Douglas Bag gas input port being plugged to ensure no external air entered the breathing circuit. In addition, SpO_2_ was monitored via portable finger pulse oximeter sensor (TORONTEK‐H50) by a lab assistant before and after their washroom break to ensure no significant changes in SpO_2_ occurred.

### 2.4 ECG morphology and conduction abnormalities

To assess electrophysiological characteristics (rate, rhythm, waveform amplitudes, durations, and intervals) of the ECG, cardiac cycles (40–70) were over‐laid and aligned with the R‐wave.

The aggregate was then analyzed using automated software (ECG analysis module; Chart Pro 8.3.1). To assess the occurrence of bradycardia and any conduction abnormalities (arrhythmias), data were extracted during the 30 s immediately preceding apneas, throughout the apnea attempts, and immediately following volitional breakpoint. To account for variation in apnea duration, HR data from the final 10 cardiac cycles of each apnea were analyzed in relation to the preceding baseline period. A cardiologist (SVD) blinded to the normoxic and hypoxic conditions identified and classified any conduction abnormalities from ECG waveforms during both free‐breathing conditions, during apnea, and following volitional breakpoint.

### 2.5 Heart rate variability analysis

Heart rate variability (HRV) analysis was performed to assess the effect of hypoxia on nonlinear cardiac autonomic modulation during free breathing. HRV analysis was not performed during apnea due to the short duration of the maneuvers and apnea‐induced instability of cardiac autonomic control. Raw ECG data were obtained from a 5‐min free‐breathing period for each time point (normoxia, AHX, and HX5) and exported to HRV analysis software (Kubios HRV Premium 3.1 k, Kubios Oy). Each 5‐min recording period underwent automatic QRS detection based on the Pan‐Tompkins algorithm (Pan & Tompkins, 1985), with additional manual review of the data to ensure accuracy and detection of ectopic beats. Ectopic beats were detected using threshold‐based median filtering, which excluded beats differing by more than 20% from their local average. Ectopic beats were further corrected via cubic spline interpolation, and data were detrended via a linear detrend function. RR interval data were transformed for spectral analysis using the Lomb–Scargle periodogram with smoothing at 0.02 Hz. Frequency bands used for spectral analysis were as follows: very low frequency (0–0.04 Hz); low frequency (0.04–0.15 Hz); high frequency (HF) (0.15–0.4 Hz). However, very low‐frequency analysis was omitted due to the time requirements required to achieve accurate readings (Electrophysiology Task Force of the European Society of Cardiology & the North American Society of Pacing & Electrophysiology, 1996). Normalized low‐frequency and high‐frequency power were calculated as follows:

$$\mathrm{Low}\mathrm{Frequency}\nu =\frac{\mathrm{LF}}{\left(\mathrm{LF}+\mathrm{HF}\right)}\times 100\%;\mathrm{High}\mathrm{Frequency}\nu =\frac{\mathrm{HF}}{\left(\mathrm{HF}+\mathrm{LF}\right)}\times 100\%$$

HRV metrics were chosen as recommended by the HRV Task Force Guidelines for short‐term HRV recordings in time and frequency domains (Electrophysiology Task Force of the European Society of Cardiology & the North American Society of Pacing & Electrophysiology, 1996), collected time‐domain measures of HRV included mean RR interval, standard deviation of the RR interval, the root of the squared mean of successive differences (RMSSD), and the percentage of RR intervals with subsequent difference >50 ms (pNN50). Frequency‐domain contributions of power (low frequency, high frequency, and total power) were analyzed.

### 2.6 Statistical analysis

Cardiorespiratory parameter statistical analyses were performed using Sigma Stat 3.13 (Systat Software). Continuous variables are reported as mean ± standard deviation. Differences in cardiovascular data between periods (normoxia vs. hypoxia 20 min [AHX] vs. hypoxia 5 h [HX5]) and between conditions (free breathing vs. end‐inspiratory vs. end‐expiratory) were assessed using two‐way repeated measures ANOVAs. HRV statistical analysis was performed using GraphPad Prism 8.4.2 (GraphPad Software). Prior to statistical analysis, HRV data were tested for normality (Shapiro–Wilk); datasets violating normality were natural‐logarithm transformed except for pNN50 due to values of zero. Differences in HRV parameters between collection periods then were assessed using one‐way RM‐ANOVA with Geisser‐Greenhouse's sphericity correction. Post hoc analysis (Holm–Sidak method) was conducted when the main effects were significant. Statistical significance was set at *p* ≤ 0.05.

## 3 RESULTS

Nineteen of 22 participants successfully completed the full HX5 period, with three participants unable to reach HX5 due to exhibiting hypoxia‐induced symptoms (headache and nausea). The normoxia and AHX data from these three participants were included for the normoxia and AHX analyses. For the results section values are reported in the order of normoxia, AHX, and HX5, respectively, unless specifically stated. Occurrences of arrhythmias in participant traces were subdivided into two distinct categories: (a) sinus pause or arrest; and (b) atrial ectopy or ectopic rhythms (see Table 2 for participant breakdown of arrhythmias).

### 3.1 Cardiorespiratory responses and conduction abnormalities during free breathing

Participant characteristics, basal cardiovascular function, ECG morphology, and ECG conduction abnormalities identified as arrhythmias during free breathing for each condition are shown in Tables 1 and 2. Resting HR was elevated during AHX (78 ± 10 bpm; *p* < 0.001 vs. normoxia) and HX5 (80 ± 12 bpm; *p* < 0.001 vs. normoxia) compared to normoxia (68 ± 10 bpm). In contrast MAP, SBP, and DBP increased from normoxia (83 ± 8, 113 ± 12, 68 ± 8 mmHg) to AHX (88 ± 9, 121 ± 13, 72 ± 9 mmHg; all *p* < 0.001 vs. normoxia), but returned to normoxic values by HX5 (83 ± 14, 110 ± 23, 83 ± 14 mmHg). ECG analysis indicated that P‐wave duration, P‐wave amplitude, and QRS duration were unchanged throughout the protocol (Table 1). However, PR‐Interval was shortened at AHX (148 ± 31 ms, *p* = 0.045) and HX5 (142 ± 32 ms; *p* < 0.05) versus normoxia (153 ± 29 ms). R‐wave and T‐wave amplitudes were also depressed at AHX and HX5 compared to normoxia.

**TABLE 1:** Demographic and cardiovascular function during free breathing during normoxia, after 20 min of hypoxia, and after 5 h of hypoxia exposure

	Normoxia *N* = 22 (F = 11)	Hypoxia ~20 min *N* = 22 (F = 11)	Hypoxia ~5 h *N* = 19 (F = 9)
Subject demographics			
Age (years)	25 ± 5	–	–
Height (m)	1.70 ± 0.08	–	–
Weight (kg)	67 ± 9	–	–
BMI (kg/m^2^)	23.3 ± 1.9	–	–
Cardiovascular function^a^			
Systolic pressure (mmHg)	113 ± 12	121 ± 13*	110 ± 23
Diastolic pressure (mmHg)	68 ± 8	72 ± 9*	68 ± 18
Cardiac output (L/min)^b^	6.3 ± 1.4	7.1 ± 1.8*	6.0 ± 1.2^†^
Total peripheral resistance^b^	14 ± 4	13 ± 4	15 ± 4
ECG metrics			
P‐wave duration (ms)	87 ± 25	87 ± 26	87 ± 22
P‐wave amplitude (mV)	0.12 ± 0.05	0.12 ± 0.04	0.12 ± 0.05
PR‐interval (ms)	153 ± 29	148 ± 31	142 ± 32*, †
QRS duration (ms)	90 ± 20	92 ± 16	93 ± 16
QTc (ms)^c^	430 ± 19	431 ± 15	437 ± 15
R‐wave amplitude (mV)	1.47 ± 0.56	1.36 ± 0.54*	1.36 ± 0.5*
T‐wave amplitude (mV)	0.35 ± 0.17	0.27 ± 0.13*	0.27 ± 0.15*

^a^ Shading was initially used to differentiate the header rows in the table.

^a^ Heart rate (bpm) and mean arterial pressure (mmHg) during free breathing are shown in Table 3.

^b^ Values calculated using Model Flow.

^c^ Framingham correction (QT + 0.154 × (1 − RR)).

* Significantly different from Normoxia; *p* < 0.05.

^†^ Significantly different from Hypoxia ~20 min; *p* < 0.05.

**TABLE 2:** ECG conduction abnormalities identified during free breathing and voluntary apnea

Participant #	Free Breathing			End‐Inspiratory Apnea			End‐Expiratory Apnea		
	Normoxia *N* = 22 (F = 11)	Hypoxia ~20 min *N* = 22 (F = 11)	Hypoxia ~5 h *N* = 19 (F = 9)	Normoxia *N* = 22 (F = 11)	Hypoxia ~20 min *N* = 22 (F = 11)	Hypoxia ~5 h *N* = 19 (F = 9)	Normoxia *N* = 22 (F = 11)	Hypoxia ~20 min *N* = 22 (F = 11)	Hypoxia ~5 h *N* = 19 (F = 9)
# of participants with identified abnormalities^a^	3	2^b^	2	1	3	2	4^b^	5	3
Participant ID									
1	–	–	–	–	–	–	–	–	–
2	–	–	–	–	–	–	–	–	–
3	–	–	^c^	–	–	^c^	–	–	^c^
4	–	–	–	D	–	–	–	–	–
5	–	–	–	–	–	–	–	–	–
6	–	–	–	–	–	–	–	–	–
7	–	–	–	–	–	–	–	–	–
8	–	–	–	–	F	G	A,F	F	G
9	–	–	–	–	–	–	–	–	–
10	–	–	–	–	–	–	D	D	–
11	–	–	^c^	–	–	^c^	–	–	^c^
12	–	–	–	–	–	G	–	–	–
13	B	B,C	A	–	–	–	–	C	–
14	A	A	–	–	A	–	–	–	A
15	–	–	–	–	–	–	–	–	–
16	D	–	–	–	–	–	–	–	–
17	–	–	–	–	–	–	–	–	–
18	–	–	–	–	–	–	C	C	–
19	–	–	^c^	–	–	^c^	–	–	^c^
20	–	–	–	–	–	–	–	–	–
21	–	–	E	–	E	–	E	E	E
22	–	–	–	–	–	–	–	–	–

Cardiac abnormality classification: (A) Ectopic Atrial Foci, (B) Wandering Atrial Pacemaker, (C) Premature Atrial Contraction, (D) Junctional Rhythm, (E) Premature Ventricular Contraction, (F) Sinus Pause/Arrest, (G) Sinus Pause w. Atrial Escape Beat. Shading was initially used to differentiate the header rows in the table.

^a^ Conduction abnormalities were identified from a standard Lead II trace. ECG assessment was carried out by a cardiologist (SVP) who was blinded to group and condition. All conduction abnormalities associated with voluntary apnea occurred immediately preceding or following break‐point.

^b^ Indicates that at least one individual during that condition was identified with multiple arrhythmic events.

^c^ Indicates that test was terminated prior to the respective time point (*N* = 3 at 5 h hypoxia exposure).

The occurrence of arrhythmias across the free‐breathing and apnea trials (both end‐inspiratory and end‐expiratory) is broken down for each participant in Table 2. However, summaries of arrhythmias identified and additional notes of interest are included in the following sections. Three individuals exhibited ectopic events during normoxic free breathing (ectopic atrial foci, wandering atrial pacemaker, and junctional rhythm). Two of these individuals still had ectopic events during AHX (ectopic atrial foci, premature atrial contraction), whereas a separate individual developed premature atrial contractions during AHX. Only one participant exhibited premature ventricular contractions at HX5.

### 3.2 HRV analysis during free breathing

**FIGURE 1:**
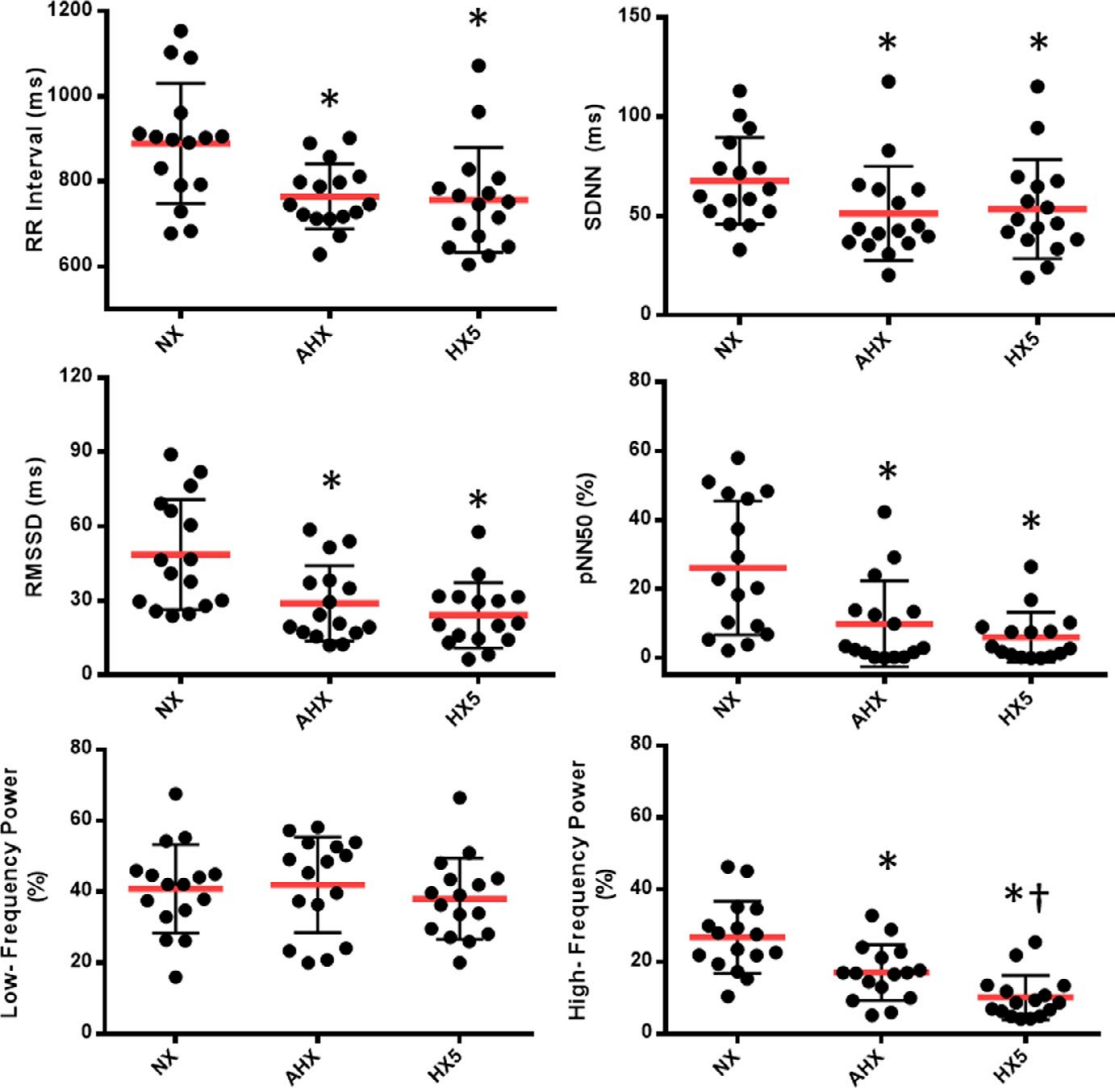
Graphical Representation of HRV metrics assessed during free‐breathing conditions under normoxia (denoted as “NX”), hypoxia at 20 min (AHX), and hypoxia at 5 h (HX5). HRV Metrics broken down into separate panels that examine RR Interval, time domain (SDNN, RMSSD, pNN50) and frequency domain (low‐frequency and high‐frequency power) analyses. AHX and subsequent HX5 had no effect on SDNN and low‐frequency power. RR interval and RMSSD decreased under AHX (*p* < 0.001 vs. normoxia) and HX5 (*p* < 0.001 vs. normoxia). pNN50 decreased at AHX (*p* < 0.05 vs. normoxia) and HX5 (*p* < 0.01 vs. normoxia). High‐frequency power decreased at AHX (*p* < 0.01 vs. normoxia) and HX5 (*p* < 0.001 vs. normoxia; *p* < 0.05 vs. AHX). *Significant difference compared to normoxia, ^†^Significant difference compared to AHX

HRV analysis for each free‐breathing condition is represented in Figure 1. Sixteen participants (8 males, 8 females) completed the protocol with all necessary HRV measurements taken. Two male participants were excluded due to exhibiting a high proportion of ectopic beats (>10%), whereas one female participant was excluded due to an insufficient ECG signal during collection periods. Mean RR interval decreased between normoxia (889.5 ± 140.8 ms) and AHX (764.7 ± 76.1 ms) (*p* < 0.001) as well as between normoxia and HX5 (756.6 ± 123.3 ms) (*p* < 0.001). Natural log‐transformed standard deviation of the NN interval (SDNN) showed no difference between normoxia (4.168 ± 0.326 log ms), AHX (3.853 ± 0.420 log ms), or HX5 (3.883 ± 0.463 log ms). Natural log‐transformed root mean squared of successive RR interval differences (RMSSD) decreased from normoxia (3.785 ± 0.460 log ms) to AHX (3.241 ± 0.5144 log ms) (*p* < 0.01), and remained decreased through HX5 (3.042 ± 0.577 log ms) (*p* < 0.001); there was no difference between AHX and HX5.

Natural log‐transformed percent HF power decreased from normoxia (3.218 ± 0.392 log percent) to AHX (2.72 ± 0.522 log percent) *p* < 0.001), further decreasing at HX5 (2.159 ± 0.5492 log percent) (*p* < 0.001 vs. normoxia; *p* < 0.01 vs. AHX). Normalized HF (HFnu) also decreased from normoxia (39.84 ± 13.72%) to AHX (29.35 ± 13.82%) (*p* < 0.01), remaining decreased at HX5 (20.81 ± 10.01%) (*p* < 0.001 vs. normoxia) and further decreasing from AHX (*p* = 0.043 vs. AHX).

### 3.3 Cardiovascular responses and conduction abnormalities during volitional apnea

**FIGURE 2:**
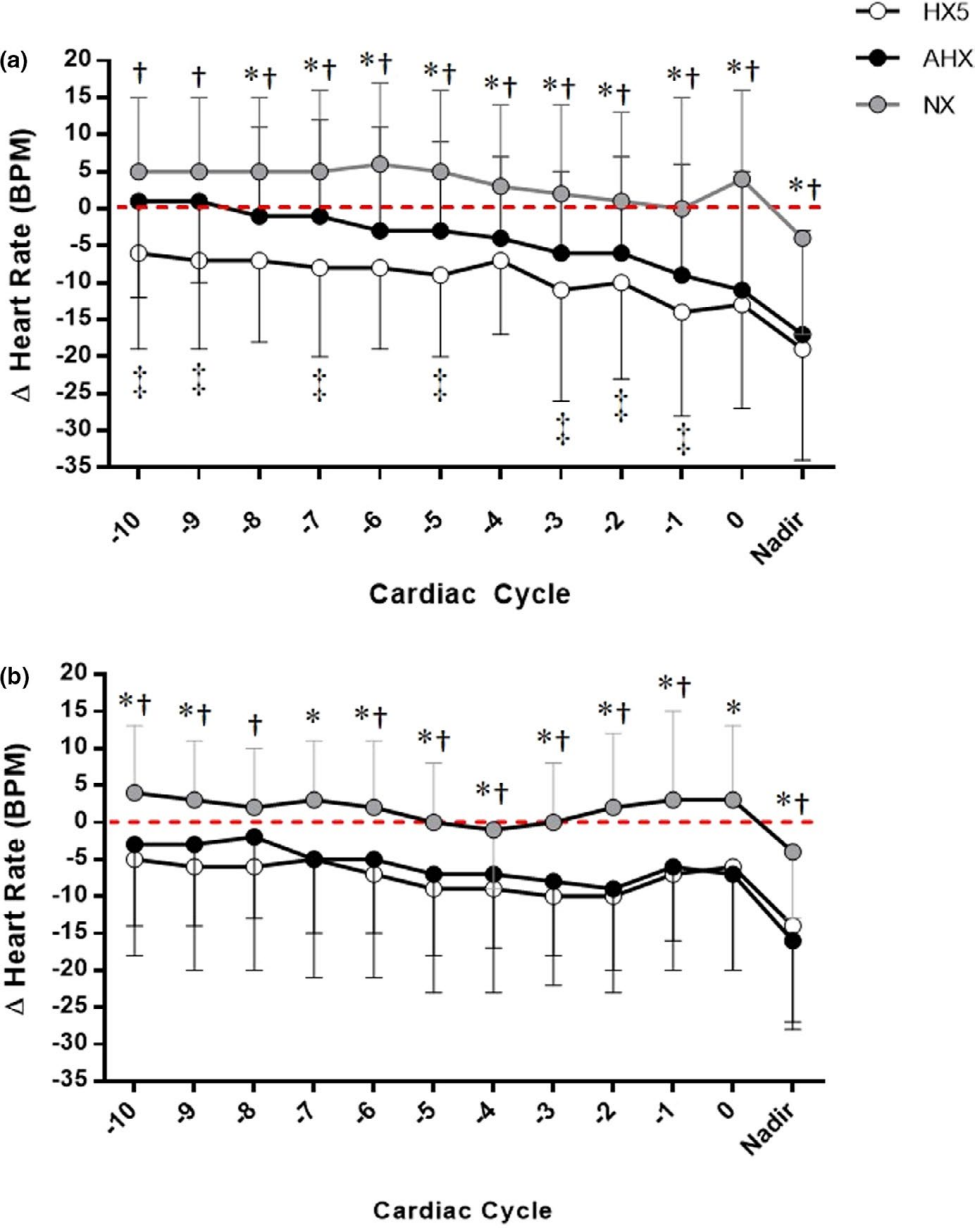
Responses to apnea in Participants under normoxia ([NORMOXIA] n.22; gray circle), and hypoxia following 20 min ([AHX] n.22; black circle) and 5 h ([HX5] n. 19; white circle) exposure. (a) Absolute bradycardia response to maximal end‐expiratory apnea attempt (at functional residual capacity). (b) Absolute bradycardia response to maximal end‐inspiratory apnea attempt. All data have been aligned to break‐point and the last 10 cardiac cycles have been plotted. The mean nadir responses are also identified. A significant bradycardia response was observed in participants under hypoxia exposure (both AHX and HX5) compared to normoxia condition, though no difference was observed between end‐inspiratory and end‐expiratory attempts. end‐expiratory showed several significant differences between AHX and HX5. *Cardiac cycle or nadir significantly different from normoxia compared to 20 min of hypoxia exposure, *p* < 0.05; ^†^Cardiac cycle or nadir significantly different between normoxia compared to 5 h of hypoxia exposure, *p* < 0.05. ^‡^Cardiac cycle or nadir significantly different from 20 min of hypoxia exposure compared to 5 h of hypoxia exposure, *p* < 0.05

Participant cardiovascular responses and ECG conduction abnormalities identified as arrhythmias during apnea for each condition are shown in Tables 2, 3 and Figure 2. Overall end‐expiratory apnea durations were shorter compared to end‐inspiratory attempts (Main Effect *p* < 0.001). Expectedly, both end‐inspiratory and end‐expiratory apnea durations were reduced at AHX and HX5, although hypoxia apneas resulted in greater reductions in SpO_2_ (Table 2). Both end‐inspiratory and end‐expiratory apneas also resulted in bradycardia that was larger during AHX and HX5 (Figure 2). However, the bradycardia occurring during end‐expiratory attempts was similar to end‐inspiratory attempts (main effect = 0.194). The peak pressor response MAP occurring during apnea was comparable between apnea types and conditions (end‐inspiratory apneas: normoxia +23 ± 12 mmHg, AHX +23 ± 15 mmHg), and HX5 (20 ± 22 mmHg; end‐expiratory apneas normoxia +19 ± 28, AHX +24 ± 17, and HX5 +26 ± 22 mmHg).

**TABLE 3:** SpO_2_, cardiovascular measures, and apnea duration during free breathing and voluntary apnea

	Free Breathing			End‐Inspiratory Apnea			End‐Expiratory Apnea		
	Normoxia *N* = 22 (F = 11)	Hypoxia ~20 min *N* = 22 (F = 11)	Hypoxia ~5 h *N* = 19 (F = 9)	Normoxia *N* = 22 (F = 11)	Hypoxia ~20 min *N* = 22 (F = 11)	Hypoxia ~5 h *N* = 19 (F = 9)	Normoxia *N* = 22 (F = 11)	Hypoxia ~20 min *N* = 22 (F = 11)	Hypoxia ~5 h *N* = 19 (F = 9)
SpO_2_ (%)^a^	98 ± 2	80 ± 4*	78 ± 3*	95 ± 4	74 ± 8*	71 ± 7*	94 ± 4	72 ± 10*	70 ± 6*
Heart rate (bpm)^a^	68 ± 10	78 ± 10*	80 ± 12*	64 ± 12	62 ± 10^§^	65 ± 14^§^	63 ± 15	61 ± 15^§^	60 ± 15^§^
Mean arterial pressure (mmHg)^a^	83 ± 8	88 ± 9*	83 ± 14	106 ± 15^§^	109 ± 19^§^	111 ± 17^§^	101 ± 12^§^	111 ± 20^§^	108 ± 20^§^
Apnea duration (s)	–	–	–	46 ± 30	33 ± 13*	35 ± 17	30 ± 19^‡^	19 ± 8^‡^	22 ± 10^‡^

^a^ End‐inspiratory and end‐expiratory values represent nadir response (SpO_2_ nadir and peak MAP response during apneas were obtained 15–20 s postvolitional breakpoint while).

* Significantly different from Normoxia; *p* < 0.05.

^†^ Significantly different from Hypoxia ~20 min; *p* < 0.05.

^‡^ Significantly different from respective end‐inspiratory apnea maneuver at same time point; *p* < 0.05.

^§^ Significantly different from free breathing at respective condition; *p* < 0.05.

The performance of end‐inspiratory apnea did not produce any notable changes in the incidence of arrhythmogenesis under both normoxia and hypoxia conditions. Only one individual exhibited a junctional rhythm during normoxia end‐inspiratory apnea, whereas three individuals exhibited arrhythmic events during AHX (ectopic atrial foci, sinus pause/arrest, premature ventricular contraction). One of the participants who developed a PVC during the end‐inspiratory attempt at AHX also had the lowest recorded SpO_2_ (nadir −28% following volitional breakpoint) alongside the longest apnea durations (~69 s). Two of 19 participants exhibited premature ventricular contractions or sinus pause with atrial escape during end‐inspiratory apnea at HX5. End‐expiratory apneas during normoxia resulted in arrhythmias for four participants (ectopic atrial foci, premature atrial contraction, junctional rhythm, premature ventricular contraction, sinus pause/arrest) which increased to five participants (premature atrial contraction, junctional rhythm, premature ventricular contraction, sinus pause/arrest) at AHX. At HX5, however, only three participants developed events during their end‐expiratory apnea attempts (ectopic atrial foci, premature ventricular contraction, sinus pause with atrial escape beat).

## 4 DISCUSSION

The goal of this study was to investigate whether vagal mediated bradycardia or transient arrhythmias previously observed during volitional apnea at altitude (several days residency at 5050 and 4350 m [Busch et al., 2018; Busch et al., 2020]) become evident during shorter term hypoxia exposure. Our findings demonstrated an attenuation of spectral high‐frequency power and pNN50 during hypoxic (SpO_2_ ~80%) free breathing which suggests a reduction in the vagal influence/increase in sympathetic influence on the heart during free breathing during hypoxia. Contrasted with the bradycardia observed during hypoxic apnea, this reinforces the hypothesis that cessation of ventilation unmasks greater chemoreflex mediated vagal drive during hypoxia which is suppressed during free breathing. Contrary to our second hypothesis, there was minimal change in the incidence of arrhythmogenesis between normoxic and hypoxia conditions.

### 4.1 ECG and HRV response to hypoxia during free‐breathing conditions

During the early stages of hypoxia (specifically AHX) participants showed a heightened basal HR which corresponded to the observed decrease in pNN50 and HF%. In addition, HX5 saw a shorter P‐R interval and QTc duration. Though still within the normal physiological range, these data support a sympathetic dominance during free breathing which agrees with previous findings that larger reductions in FiO_2_ appear to further reduce HF (Guger et al., 2008; Iwasaki et al., 2006; Rupp et al., 2013). We also observed blunting of R‐ and T‐wave amplitudes which we and others have demonstrated during prolonged (Busch et al., 2018) and acute hypoxia (Coustet et al., 2015).

### 4.2 Cardiac response to apnea during normoxia and hypoxia

There was an overall greater bradycardia response for both end‐inspiratory and end‐expiratory apneas under hypoxic conditions when compared to normoxia apneas. Bradycardia during apnea occurs through heightened vagal tone associated with the mammalian diving reflex (Bain et al., 2018; Ferrigno et al., 1986; Liner, 1994) as an adaptive response to conserve arterial oxygen content. However, the degree of bradycardia response is reported to be minimal during normoxic conditions in untrained individuals (Busch et al., 2019; Heusser et al., 2009). Accordingly, bradycardia was only observed during hypoxia periods despite overall shorter apnea durations. This significant bradycardia can be explained through hypoxia‐induced peripheral chemoreceptor activation and a subsequently heightened vagal drive (Burgh Daly et al., 1979; Burgh Daly & Scott, 1962). Such heightened chemoreceptor activation during hypoxia is masked during free breathing by pulmonary reflexes that inhibit both parasympathetic (Burgh Daly, 1997) and sympathetic activity (Busch et al., 2020). Yet with apnea attempts within ranges of normal tidal volumes (especially during the end‐expiratory attempts), chemoreflex‐mediated vagal drive becomes more prominent leading to more pronounced bradycardia. While the decrease in heart‐rate observed during hypoxic apneas was significant (−15 to −20 beats per min), it is worth noting that this is less than previously observed following apnea during prolonged hypoxia at apnea following 5–10 days at 5050 m (−30 to −35 bpm; (Busch et al., 2018; Busch et al., 2020). Thus, carotid body sensitization with prolonged hypoxia likely plays a role in the magnitude of the bradycardia response. Though not the focus of this study, we acknowledge that the heightened MAP during each attempt may facilitate further bradycardia via baroreceptor loading (Heusser et al., 2009), though the relatively short period in our participants’ apnea attempts would likely result in the baroreflex not being a contributing factor, as the baroreflex‐mediated suppression of HR would be expected to be similar.

Previously, we identified an incidence of bradyarrhythmias during normoxic end‐expiratory apnea of 21% (3/14 participants) that substantially increased to 79% (11/14 participants) at altitude (5050 m) (Busch et al., 2018); we further confirmed an elevated incidence of bradyarrhythmia at altitude in a separate cohort (62%; 8/13 participants at 4350 m, [Busch et al., 2020]). In this study, we identified a similar incidence of arrhythmia during normoxic end‐expiratory apnea of 18% (4/22 participants), with the incidence during normoxic end‐inspiratory apnea being 5% (1/22 participants). However, the incidence of arrhythmia did not increase appreciably during acute hypoxia (9% and 23% for inspiratory and expiratory apneas) or following 5 h of hypoxia (9% and 16% for inspiratory and expiratory apneas). We do not believe the discrepancy in the incidence of arrhythmias between this study and our previous work is due to a difference in the severity of the hypoxia. In the current protocol, we specifically targeted a SpO_2_ which matched that measured during our altitude studies (Busch et al., 2018; Busch et al., 2020). We interpret these findings to indicate that acclimatization to prolonged hypoxia, and carotid body sensitization, is a critical component of the development of apnea‐induced arrhythmia. This is in keeping with the previously observed relationship between the incidence of arrhythmia and the gain of the hypoxic ventilatory response, mediated by the peripheral chemoreflex (Busch et al., 2018).

Although the exact mechanism driving bradyarrhythmogenesis during prolonged hypoxia remains unclear, we have previously attributed this to the competing and overall conflicting relationship between sympathetic and parasympathetic innervation within the heart (Burgh Daly, 1997; Kollai & Koizumi, 1979; Paton et al., 2005; Somers et al., 1992). Similar occurrence of cardiac arrhythmias has been observed during cold‐water immersion (i.e., including apnea) (Shattock & Tipton, 2012), where a high degree of both sympathetic (via the cold shock response) and parasympathetic (via the diving response) activity occurs concurrently. Though the occurrence of apnea‐induced autonomic conflict during cold water immersion would likely produce a greater degree of parasympathetic/ sympathetic activation than that seen in this study (i.e., via additional trigeminal stimulation); we believe that conflicting cardiac autonomic activity may also exist when significant chemoreflex activation occurs (Kollai & Koizumi, 1979). For example, the previously documented arrhythmias in highly trained breath‐hold divers during maximal static apnea attempts spanning several minutes (HanseI et al., 2008; Lemaitre et al., 2005; Lindholm & Lundgren, 2009) occur alongside a high degree of vagal (Lemaitre et al., 2005; Perini et al., 2008; Schagatay & Andersson, 1998) and sympathetic (Dujic et al., 2008; Heusser et al., 2009) tone that is driven primarily through hypercapnic/hypoxic strain. However, chronic hypoxia exposure is likely to impart additional stress versus the relatively shorter apnea durations reported in professional apneists (Lemaitre et al., 2005; Lindholm & Lundgren, 2009). Given the previously stated morphological changes that carotid bodies exhibit in response to chronic hypoxia (Arias‐Stella & Valcarcel, 1976; Lahiri et al., 2000; Lopez‐Barneo et al., 2008; Wang et al., 2008), other such adaptations along the chemoreflex pathway that facilitate heightened sensitivity to hypoxia may contribute to the arrhythmogenesis observed at altitude. The inability of our apneic model to evoke a change in the incidence of arrhythmic events following 5 h of hypoxia further supports the argument that a time‐dependant change in peripheral chemoreflex may exist following several days of hypoxia exposure.

### 4.3 Considerations and limitations

This study aimed to examine peripheral chemoreceptor sensitization during moderate hypoxia exposure, as observed in previous human (Lahiri et al., 2000; Sato et al., 1992) and animal studies (Bisgard et al., 1986; Nielsen et al., 1988), and its influence on the incidence of bradyarrhythmogenesis during apnea. However, we recognize that other overlapping mechanisms may affect sympathovagal influence to the heart and potentially contribute to the development of apnea‐induced bradyarrhythmogenesis. Central chemoreflex CO_2_ activation, and its interaction with the peripheral chemoreflex, has been thoroughly studied with regard to its influence on the ventilatory response to hypoxia (as reviewed previously [Dempsey et al., 2014; Guyenet, 2014; Powell, 2007]). While direct central nervous system (CNS) exposure to acute hypoxia alone does not alter ventilatory response when isolated (Neubauer et al., 1990; Van Beek et al., 1984), an interdependent relationship between peripheral and central chemoreceptors may be facilitated through chemosensitive organs within the brain. Chemical‐sensitive organs within the CNS, such as the CO_2_‐sensitive retrotrapezoid nucleus, may go beyond solely mediating central chemoreflex response to CO_2_/H^+^ to also integrate or respond to increase carotid sinus nerve input, thus acting as a link to other regulatory systems in the CNS (Guyenet et al., 2010). Other CNS oxygen‐sensitive organs may also contribute, though their interaction with chemosensory inputs is unclear (Powell et al., 2009). Such interdependence between central and peripheral pathways continues to be a highly contested topic as to whether the results produce additive (Duffin & Mateika, 2013), hypoadditive (Wilson & Day, 2013) or hyperadditive (Teppema & Smith, 2013) effects on the ventilatory gain response to hypoxia. We propose the peripheral chemoreceptor sensitization to be a potential candidate for bradyarrhythmogenesis at altitude, as shown previously (Busch et al., 2018). We, however, also acknowledge that a more complex interaction between overlapping central and peripheral chemoreflex pathway sensitization may exist under chronic hypoxia that translates not only to ventilatory acclimatization at altitude, but also heightened autonomic innervation to the heart during apnea. Further research is required to both verify and delineate such underlying mechanisms.

The two separate apnea maneuvers (end‐inspiratory and end‐expiratory) performed across all conditions explored the effects of static lung volume on the development of bradyarrhythmias. Due to the inhibitory effects of pulmonary stretch on both respiratory and cardiovascular control centers (Busch et al., 2019; Macefield & Wallin, 1995; Muxworthy, 1955); we hypothesized that higher lung volumes would result in both longer apnea duration and a reduced incidence of arrhythmogenesis. Our findings indeed showed shorter apnea durations for end‐expiratory maneuvers across conditions, whereas HR responses were similar across maneuvers. Taken together, these findings argue in favor of the end‐expiratory maneuvers being an overall greater physiological stressor compared to the end‐inspiratory maneuvers. Although it cannot be statistically proven, more individuals tended to exhibit arrhythmias during the end‐expiratory portion. However, the lack of change in end‐expiratory arrhythmogenesis between normoxia and hypoxia protocols further suggests that the rate of change in cardiovagal drive plays a lesser factor in the incidence of arrhythmogenesis. Our participants showed comparable degrees of desaturation and apnea duration to our previous observations in Lowlanders at 5050 m (where 11 of the 14 individuals tested exhibited varying degrees of arrhythmogenesis), with the bradycardia responses in our earlier findings (Busch et al., 2018; Busch et al., 2020) being overall lower than what was observed in this study.

Several limitations must be addressed with regard to this study design. The first being the lack of a “control” 5 hour normoxia exposure in order to account for nonspecific effects of the oronasal facemask and the semi‐recumbent position participants was seated in. As previously demonstrated by Askanazi et al. (1980), the use of oronasal facemask alters the human ventilatory pattern through changes in respiratory dead space that influence minute ventilation and tidal volume, though there appears to be no difference in O_2_ consumption or CO_2_ production. Within the context of our study, we have no reason to suspect this would significantly affect the incidence of arrhythmogenesis, as the apneic model removes any influence on respiratory gating while uncovering the heightened autonomic activity that would exist due to hypoxia alone. The consideration of participant position also likely has minimal effect, cardiac autonomic activity has previously been shown to not be affected by seating position in young healthy individuals (Watanabe et al., 2007). Regardless, both factors should be considered within the context of our findings, and be considered in future studies to account for these points.

Another limitation of the study design involves the use of normobaric hypoxia to replicate a similar SpO_2_ as those seen in former studies at altitude (Boos et al., 2017; Busch et al., 2018; Busch et al., 2020; Cummings & Lysgaard, 1981). This point is relevant with regard to the current discussion surrounding differences in the physiological response to normobaric versus hypobaric hypoxia (Millet & Debevec, 2020; Richalet, 2020). Although our participants were tested under normobaric hypoxia (approximate FiO_2_ range 10–11%); we believe that the lack of change in the incidence of arrhythmias compared to field studies (>4000 m) is still attributable to a duration factor (several hours vs. several days exposure) more so than the mode of hypoxia administration. In addition, we were unable to find any literature that specifically discerns a difference in either cardiac function or carotid body activation between normobaric and hypobaric hypoxia at rest, outside of possible differences in the magnitude of basal heart rate and SpO_2_ during acute exposure (Savourey et al., 2003).

## 5 CONCLUSION

In summary, the current data demonstrate that although increased vagal influence occurs during apnea following short‐term hypoxia exposure, there is no increase in the occurrence of apnea‐induced bradyarrhythmia compared to the normoxic state. Although this study does not directly measure carotid sinus activity, peripheral chemoreflex activation may be implicated in the development of apnea‐induced bradycardia. Future research is required to characterize the duration and severity of hypoxia necessary to evoke bradyarrhythmias similar to those previously reported during prolonged stays at altitude.

## CONFLICT OF INTEREST

The authors declare no conflicts of interest, financial or otherwise.

## AUTHORS’ CONTRIBUTIONS

The experiments within this study were conducted at Neurovascular Health Lab (University of Alberta, Edmonton; Canada). Co‐authors listed have contributed to either (a) Conception or design of work (SAB, LB, MS, CB, and CDS), (b) Acquisition, analysis, or interpretation of data for the work (SAB, SVD, RR, AS, LB, MS, and CB), or (c) Drafting the work or revising it critically for important intellectual content (SAB, SVD, RR, AS, LB, MS, CB, and CDS). All persons listed have qualified for authorship and approve of the final version of the manuscript. Finally, all authors listed agree to be accountable with regard to ensuring accuracy and integrity for the work currently investigated.
